# Digital Competencies and Attitudes Toward Digital Adherence Solutions Among Elderly Patients Treated With Novel Anticoagulants: Qualitative Study

**DOI:** 10.2196/13077

**Published:** 2020-01-24

**Authors:** Maximilian Herrmann, Philip Boehme, Arne Hansen, Katharina Jansson, Patrick Rebacz, Jan P Ehlers, Thomas Mondritzki, Hubert Truebel

**Affiliations:** 1 Didactics and Educational Research in Health Science Faculty of Health Witten/Herdecke University Witten Germany; 2 Research & Development, Pharmaceuticals Bayer Aktiengesellschaft Wuppertal Germany; 3 Production Planning & Logistics Johnson & Johnson Medical Gesellschaft mit beschränkter Haftung Norderstedt Germany

**Keywords:** medication adherence, eHealth, mHealth, digital health, smartphone, elderly patients, compliance, digital device, digital competencies, grounded theory, delivery of health care, diffusion of innovation

## Abstract

**Background:**

Nonadherence to medication is a driver of morbidity and mortality, and complex medication regimens in patients with chronic diseases foster the problem. Digital technology might help, but despite numerous solutions being developed, none are currently widely used, and acceptance rates remain low, especially among the elderly.

**Objective:**

This study aimed to better understand and operationalize how new digital solutions can be evaluated. Particularly, the goal was to identify factors that help digital approaches targeting adherence to become more widely accepted.

**Methods:**

A qualitative study using a conceptual grounded theory approach was conducted. We included patients aged 65 years and older who routinely took new oral anticoagulants. To generate theses about the digital competencies of the target group with daily medication intake, face-to-face interviews were conducted, recorded, and anonymized. After coding the interviews, categories were generated, discussed, and combined with several theses until saturation of the statements was reached.

**Results:**

The methodological approach led to the finding that after interviews in 20 of 77 potentially available patients, a saturation of statements was reached. The average patient’s age was 75 years, and 50% (10/20) of the subjects were female. The data identified five main coding categories—Diseases and medicine, Technology, Autonomy, Patient narrative, and Attitude toward technologies—each including positive and negative subcategories. Main categories and subcategories were summarized as Adherence Radar, which can be considered as a framework to assess the potential of adherence solutions in the process of prototyping and can be applied to all adherence tools in a holistic manner.

**Conclusions:**

The Adherence Radar can be used to increase the acceptance rate of digital solutions targeting adherence. For a patient-centric design, an app should be adapted to the individual patient’s needs. According to our results, this application should be based on gender and educational background as well as the individual physician-patient relationship. If used in a proper, individualized manner, digital adherence solutions could become a new cornerstone for the treatment of chronically ill individuals.

## Introduction

### Background

**Figure 1:**
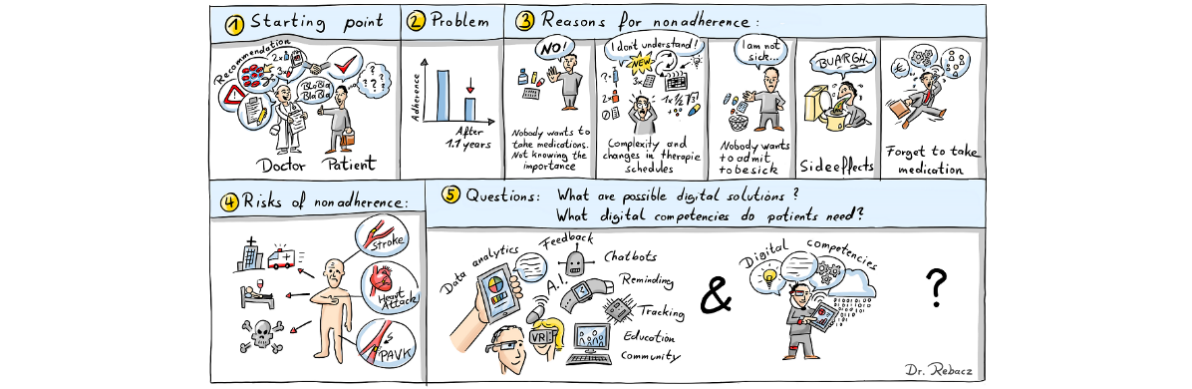
Research question: How digital competencies can influence adherence?

Nonadherence to prescribed medications is a driver of morbidity and mortality. A recent report of the World Health Organization (WHO) has shown that approximately 50% of the patients are nonadherent to their medication in developed countries, and that percentage is even higher in middle- and low-income countries [1,2]. Nonadherence to medication in case of chronic diseases has different reasons and leads to an increased risk for hospitalization and death (Figure 1).

Nonadherence usually worsens when patients with chronic diseases must adhere to complex medication regimens [3]. The total cost of nonadherence is estimated to be between US $100 and US $300 billion, and nonadherence is also responsible for more than 125,000 deaths per year in the United States alone [4-6]. For years, the research on chronic diseases has shown that in a setting of a clinical trial, adherence can be increased by using nondigital (“digital” is defined as continuous data collection and instant feedback) devices [7]. In a trial of 2 months, Laster et al [8] were able to show an increase in adherence of 12.7% for patients with glaucoma using an electronic medication alarm device, which displays the last time the bottle was opened. Rosen et al [9] also showed an increase in adherence of 15% for patients with diabetes using an electronic monitoring cap, which records the date and time the bottle was opened in a trial of 3.5 months. Despite the remarkable increase of adherence in clinical trials, these solutions did not translate into the treatment of patients. Reasons for that could be the inconvenient use of the readout by health care personnel, the delayed feedback, or the associated costs [7].

### Objectives

To better understand how new digital devices can increase adherence in a real-life scenario and why no solution has reached broad acceptance yet, we conducted a qualitative study to (1) identify factors that make digital solutions successful and (2) generate theses to understand which digital competencies patients aged 65 years and older on anticoagulants should have, to be able to use a digital solution to increase adherence. The sample selected was a population of patients that routinely took new oral anticoagulants (NOACs). Adherence to this type of medication is especially important because NOACs need to be taken daily to prevent the occurrence of thrombotic or embolic events [10,11].

## Methods

### Design

To learn how to design a digital adherence solution, we generated theses about the digital competencies of a target group with daily medication intake. Therefore, a qualitative study according to Mayring [12], with a conceptual and theoretical approach based on the Straussian *grounded theory* [13-15], was conducted in patients aged 65 years and older on anticoagulants. Face-to-face guided interviews were performed to generate theses. The responses were recorded and anonymized. Afterward, the interviews were evaluated qualitatively using the software MAXQDA (Version 13, VERBI Software, Berlin, Germany) by two independent examiners. After coding of the interviews was accomplished, categories were generated, discussed, and combined with several theses until saturation of the statements was reached.

### Recruitment of Participants

General practitioners associated with Witten/Herdecke University cooperated to identify participants for this study. Inclusion criteria comprised age (>65 years) and the intake of an NOAC. No further exclusion criteria or screening questionnaires were applied. Cooperating general practitioners contacted potential participants and asked them to volunteer. A cover letter clarified the intent of the study. The privacy policy was provided via an additional letter. A sampling procedure was performed in a probabilistic manner. All participants were informed about study details, for example, duration of the study interview or data storage policy. Participants willing to consent were asked to send a reply letter with their signed consent form in a prepaid envelope.

The Ethics Committee at Witten/Herdecke, University Faculty of Medicine, authorized the study and its ethical and legal implications (statement no. 89/2017).

### Data Collection

Data were collected from August 2017 to December 2017. Face-to-face interviews were either conducted at the participants’ home or at Witten/Herdecke University. Each interview was audio recorded with the participant’s permission. The study methods and results were reported according to the Consolidated Criteria for Reporting Qualitative Research (COREQ) [16]. The completed COREQ checklist can be found in Multimedia Appendix 1.

### Data Analysis and Statistics

To evaluate the digital competencies of the participants, an open interview guideline was created (Multimedia Appendix 2) by an expert panel with a multiprofessional background in medicine and engineering, pharma, psychology, and economics (for more details on the research team and reflexivity, see Multimedia Appendix 1). The interview guideline was previously tested in 5 randomized participants (who were subsequently not included in the results) in pilot interviews and was reviewed and revised by the expert panel. The process was supported by a literature review of recent publications on digital competencies and usability of various types of technical and digital solutions to increase adherence. These types of technical and digital solutions range from interactive notification apps, which remind the patient to take their medication, and education-based apps (eg, Transplant Hero and Incendant 360° Patient Education Suite) to smart wireless pill bottles, which alert the patient and send a message to the patient’s phone (eg, Smart Pill Bottle of AdhereTech). In addition, solutions that use an ingestible sensor to measure the intake of the medication in stomach (eg, Proteus Discover) or artificial intelligence technologies that leverage a visual recognition algorithm to monitor patient adherence (eg, AiCure) were evaluated. Finally, we assessed an intelligent and socially interactive health care robot intended to help patients with their disease management (eg, Mabu Personal Healthcare Companion). To generate theses, the conceptual approach of *Grounded Theory*, a method applicable when investigating social processes related to complex phenomena, was used (for more details on the study design, see Multimedia Appendix 1). The approach is based on the subjective experience of participants. Straussian *grounded theory* was used to analyze the generated data in the following three stages: open coding, axial coding, and selective coding. A special focus was placed on the open coding approach of the *grounded theory* method. This approach needed to be confirmed in additional theory formation and further studies. Memos were written continuously to document the conceptual and theoretical ideas that emerged when exploring the data [13-15]. Audio-recorded interviews were coded directly by two examiners without transcription by using software MAXQDA (VERBI Software; for more details on analysis and findings, see Multimedia Appendix 1). Table 1 shows 2 examples of the coding process.

The comparisons between the groups for category parameters in the quantitative and social demographic parts of the questions were achieved with the Fisher exact test. The difference was defined as statistically significant if a value of *P*<.05 was reached.

**Table 1:** Examples for creating categories.

Quote	Open coding	Axial coding (subcategory)	Selective coding (core category)
“The more I know about the disease, the more crazy obsessed I become.”	Knowledge, disease, more crazy obsessed	Increasing knowledge of a medical condition can be worrisome for the patient	Diseases and medicine
“I don’t want to know so much about my illness, because then I just worry too much about it.”	Knowledge, illness, worry too much	Increasing knowledge of a medical condition can be worrisome for the patient	Diseases and medicine
“From my point of view digital solutions are important for life-threatening conditions where I need to take a medication.”	Digital solution, life-threatening condition, medication	Digital solutions should be developed for life-threating illness	Diseases and medicine
“I do not want a medical app unless it is essential for life and absolutely necessary for my illness.”	Medical app, essential for life, necessary for illness	Digital solutions should be developed for life-threating illness	Diseases and medicine
“Digitisation can’t be stopped, but you have to be careful not to lose your independence if you rely too much on digital solutions.”	Digitization, loss of independence, reliability	Too much reliance on digital solutions can lead to loss of independence	Autonomy
“By digital solutions and a control of the intake of my medication, I would feel incapacitated.”	Digital solution, control, feel incapacitated	Too much reliance on digital solutions can lead to loss of independence	Autonomy
“Nowadays I find the constant availability due to digital solutions annoying.”	Constant availability, digital solutions, annoying	Digital solutions can promote the feeling of being surveilled	Autonomy
“I don’t want to be a puppet of digital solutions which want to control me constantly.”	To be a puppet, digital solutions, constant control	Digital solutions can promote the feeling of being surveilled	Autonomy

## Results

### Participants

A total of 77 participants were identified at family practitioners’ offices affiliated with Witten/Herdecke University. We subsequently recruited patients and performed interviews until saturation of the statements was reached (after N=20; Table 2). Participants were defined as *nonadherent* if they did not take their prescribed medication once in the last 4 weeks. Otherwise, participants were classified as *adherent*. The classification of nonadherence was based on participant self-report of their medication adherence. The average age was 75 years, and 50% (10/20) of the patients were female. Moreover, 60% (12/20) of the participants were married or lived in a partnership, 30% (6/20) were single or widowed, and 10% (2/20) lived in a more generational household. Of the 20 participants, 11 (55%) had from atrial fibrillation. In addition, 75% (15/20) took Eliquis (apixaban), 20% (4/20) took Xarelto (rivaroxaban), and 5% (1/20) took Pradaxa (dabigatran). At the time of the study, the average duration of NOAC usage was 29.3 months. Of the 20 participants, 60% (n=12) used a smartphone, 20% (n=4) used a mobile phone, 15% (n=3) used a senior mobile phone (mobile phone especially for seniors with, for example, large buttons and large display), and only 5% (n=1) had no phone. Of the 20 participants, 13 (65%) used other digital devices than a phone. In total, 60% (12/20) reported being adherent.

**Table 2:** Participant demographics.

Patient	Sex	Age (years)	Profession	Marital status	Indication	Medication	Medication before NOAC^a^	Duration on NOAC (month)	Type of phone	Other digital devices	Do you forget to take your medication?
1	M^b^	73	Mechanical engineer	S/W^c^	ST^d^, CA^e^	Xarelto	ASA^f^	36	SP^g^	—^h^	Yes
2	F^i^	83	Qualified salesperson	S/W	ST	Eliquis	Marcumar	36	MP^j^	—	No
3	F	69	Personnel administrator	S/W	Afib^k^, PM^l^, TB^m^, ST	Pradaxa	Marcumar	144	MP	PC^n^	No
4	M	72	Diploma in public administration	M/P^o^	Afib	Eliquis	Marcumar	6	SP	PC, Tablet	Yes
5	M	74	Engineer	MGH^p^	CA	Eliquis	—	24	SP	PC	No
6	M	68	IT sales staff	M/P	ST	Xarelto	—	3	SP	Tablet, PC	No
7	F	70	Hairdresser	M/P	CA, PM^q^	Eliquis	Marcumar	5	MP	—	No
8	F	76	Secretary	S/W	TB	Eliquis	Xarelto	60	SP	PC	No
9	F	88	Housewife	S/W	Afib	Eliquis	—	7	NMP^r^	—	Yes
10	F	72	Teacher	M/P	CA, valve does not work properly	Xarelto	Marcumar, Eliquis	3	SP	—	No
11	F	82	Tailor	MGH	TB	Eliquis	—	12	SMP^s^	Tablet	No
12	M	77	Postal service employee	M/P	Afib	Eliquis	ASA	4	SMP	—	Yes
13	M	68	Teacher	M/P	Afib	Eliquis	—	24	SP	Laptop	Yes
14	M	85	Electrical engineer	S/W	Afib, PM	Xarelto	Marcumar	36	SP	PC, laptop, and tracker	Yes
15	M	70	Pharma sales representative	M/P	Afib	Eliquis	—	17	SP	PC	No
16	M	72	Lawyer	M/P	Afib	Eliquis	—	24	SP	PC	Yes
17	F	76	Pharmacist	M/P	Afib	Eliquis	—	24	SP	PC, tablet, and heart rate watch	No
18	M	75	Businessman, reporter, publisher	M/P	Afib	Eliquis	—	108	SP	PC, Apple watch, and tablet	Yes
19	F	78	Industrial management assistant	M/P	Afib	Eliquis	—	9	MP	—	No
20	F	78	Childminder	M/P	Afib, CA	Eliquis	—	4	SMP	Tracker and tablet	No

^a^NOAC: new oral anticoagulant.

^b^M: male.

^c^S/W: single/widowed.

^d^ST: stroke.

^e^CA: cardiac arrhythmia.

^f^ASA: acetylsalicylic acid.

^g^SP: smartphone.

^h^No medication before NOAC/no other digital devices.

^i^F: female.

^j^MP: mobile phone.

^k^Afib: atrial fibrillation.

^l^PM: pacemaker.

^m^TB: thrombosis.

^n^PC: personal computer.

^o^M/P: married/partnership.

^p^MGH: more generational household.

^q^PM: pacemaker.

^r^NMP: no mobile phone.

^s^SMP: senior mobile phone.

**Figure 2:**
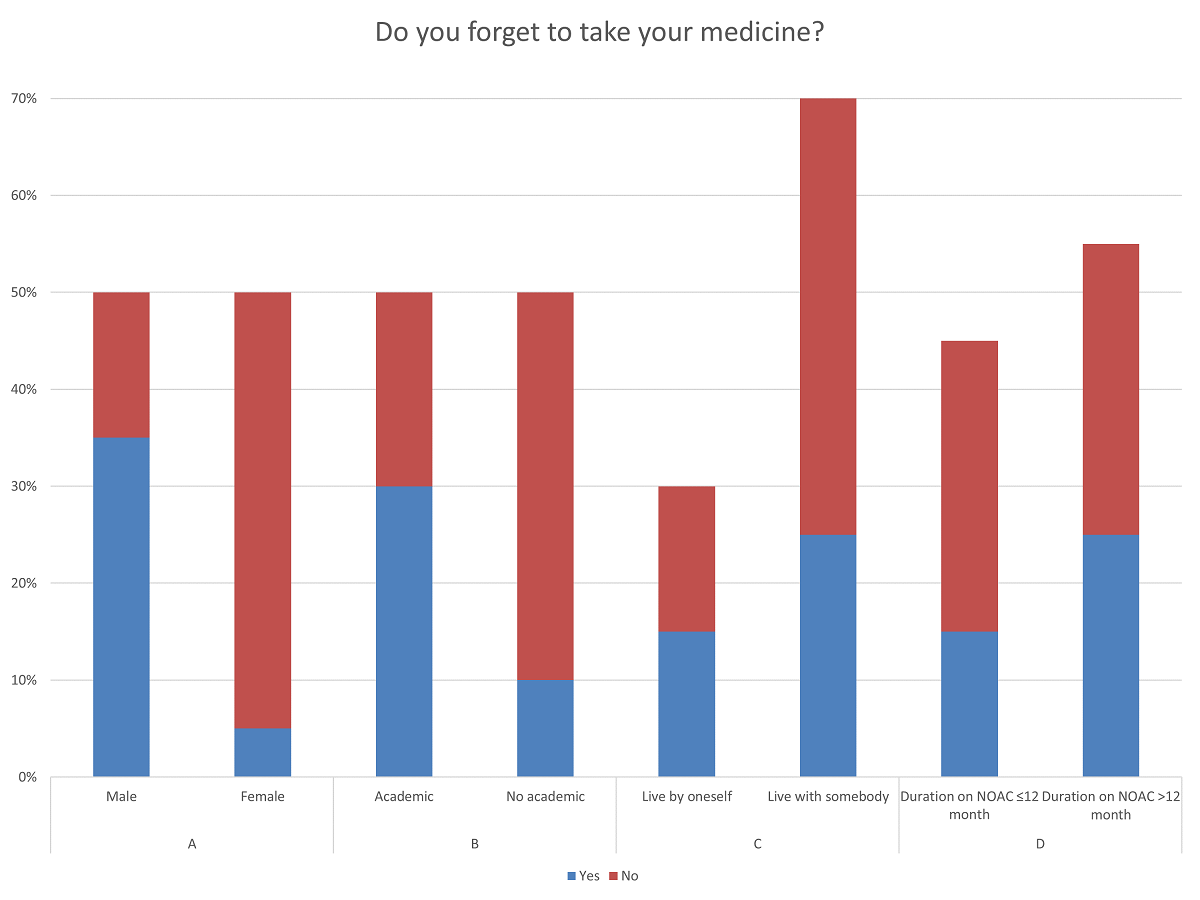
Participants-reported adherence by attributes.

As shown in Figure 2, more female participants (90%; 9/10) reported being adherent in contrast to male participants (30%; 3/10). The Fisher exact test indicated statistical evidence that female participants were significantly more likely to report being adherent than male participants (Fisher exact test, *P*_2A_=.02). The results also show that more academics (6/10, 60%) reported nonadherence than nonacademics (Figure 2). Although not statistically significant (Fisher exact test, *P*_2B_=.67 and *P*_2C_=.07), more participants (50%; 3/6) who lived by themselves reported being nonadherent than participants (36%; 5/14) who lived in company (Figure 2). Of participants taking NOACs ≤12 months, only 33% (3/9) reported nonadherence, and longer medication duration led to an increase in reporting of nonadherence (5/11, 46%; Fisher exact test, *P*_2D_=.64), as shown in Figure 2.

**Figure 3:**
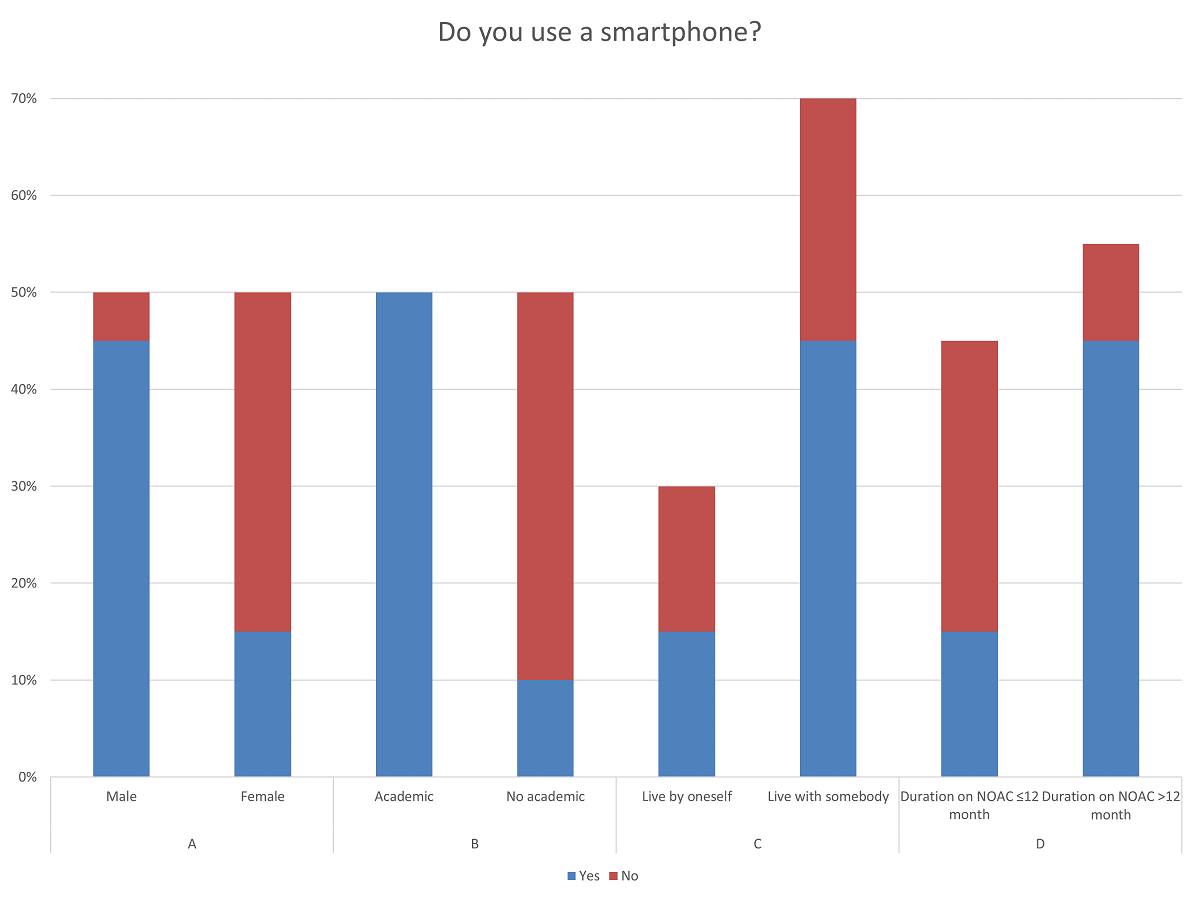
Participant smartphone use by attributes.

As shown in Figure 3, most male participants (9/10, 90%) used a smartphone, whereas fewer female participants reported using a smartphone (3/10, 30%). In Figure 3, all academics (N=10) used a smartphone in contrast to nonacademics (2/10, 20%). There is statistical evidence that male participants were significantly more likely to use a smartphone than female participants (Fisher exact test, *P*_3A_=.02) and that academics were significantly more likely to use a smartphone than nonacademics (Fisher exact test, *P*_3B_<.001). As shown in Figure 3, fewer participants (3/6, 50%) who lived by themselves used a smartphone as compared to the participants (9/14, 64%) who lived with somebody, and fewer participants (3/9, 33%) who were taking NOACs for ≤12 months used a smartphone in comparison with participants (9/11, 82%) who were taking NOACs >12 months. The last two results in Figure 3 were not statistically significant (Fisher exact test, *P*_3C_=.64; Fisher exact test, *P*_3D_=.67).

### Open Interview Results and Development of an Adherence Radar

**Figure 4:**
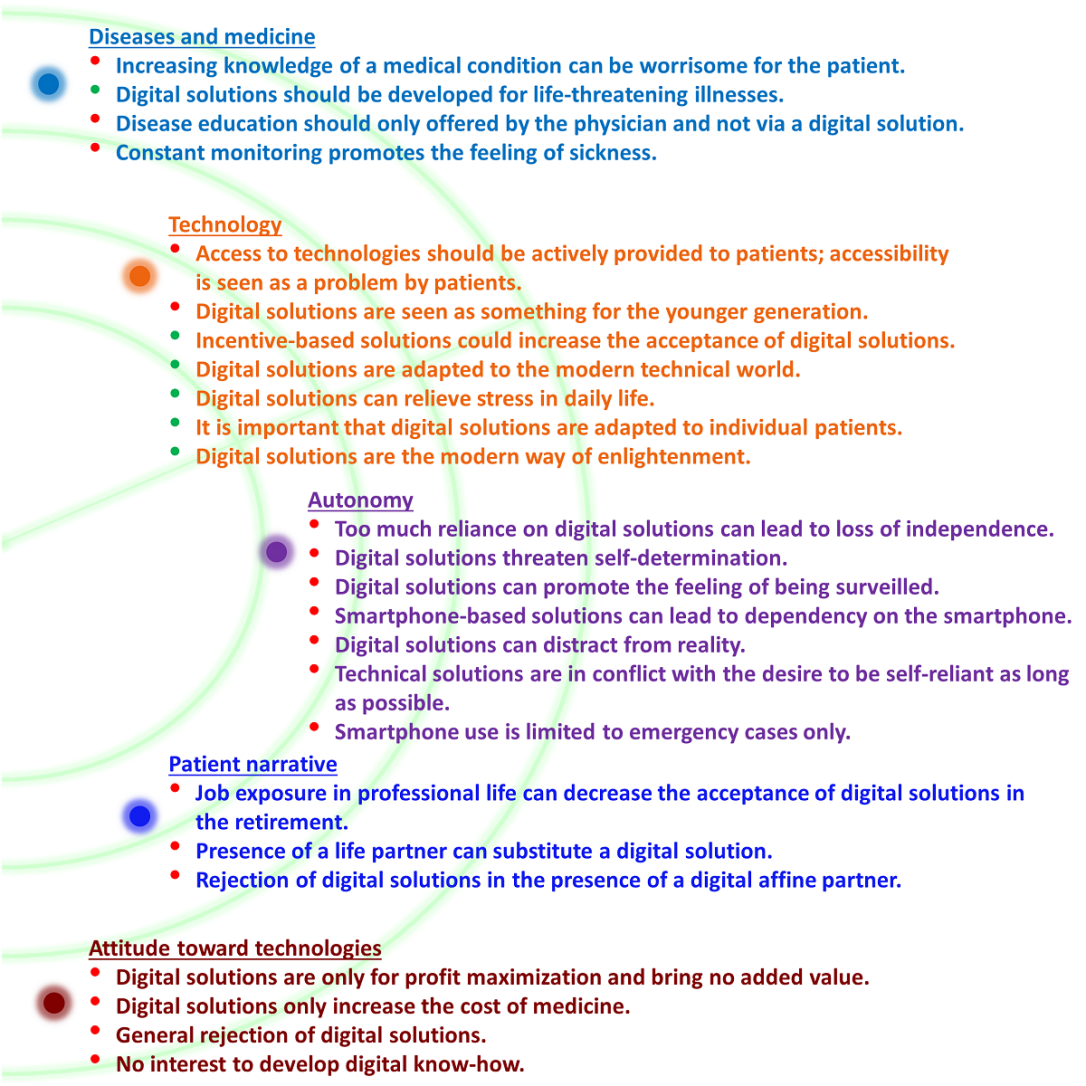
Adherence Radar: Main and subcategories. Green bullet points denote positive and red bullet points denote negative subcategories.

In open interviews, participants were asked about their general use of digital devices, especially smartphones, and their experience with these digital devices. Moreover, patients were shown different digital solutions to increase adherence. The results of the Straussian *grounded theory* approach, where we used three stages—open coding, axial coding, and selective coding—to analyze the generated data, could identify the following five main categories, and each main category includes positive and negative subcategories (Figure 4).

1. Diseases and Medicine: This category deals with the general feelings and thoughts of the patients toward diseases, for example, “The more I know about the disease, the more crazy obsessed I become.” Furthermore, this main category is about the knowledge of the patient about medicine, for example, “I feel fully informed by my physician about my medicine and do not need digital support.”
2. Technology: This category addresses, especially, the assessment and technological acceptance of the patients toward digital solutions in the health care sector, for example, “Digital solutions in the health care sector are good, they are adapted to today's technical world” as well as barriers of these technologies in a daily life, for example, “In general, I am interested in new technologies but have no access.”
3. Autonomy: This category deals with the general feeling of dependency toward the digital transformation of the health care sector, for example, “Digitization is unstoppable, but you have to be careful not to lose your independence,” and with the freedom from external control or influence of digital solutions in the health care sector, for example, “I do not want to become the puppet of systems.”
4. Patient narrative: This category summarizes the general thoughts toward the use of digital solutions in the health care sector, for example, “I've had enough to do in my professional life with digital things, in my retirement I have no interest in it anymore,” and includes the willingness and interest of a digital solution when they live in a partnership, for example, “I do not need digital solutions, my partner supports me in taking my medication regularly.”
5. Attitude toward technologies: This category covers the attitude toward using a digital solution, for example, “I am no longer interested in the digital age and do not want to accept it anymore,” and the risk of misuse of such technologies in the health care sector, for example, “Digital solutions are of no use to me, they only increase the cost of medicines.”

## Discussion

### Adherence

Here, we report on a population of patients aged 65 years and older treated with NOACs. These patients were chosen because regular intake is required for sufficient protection from adverse events such as stroke or embolism. A *grounded theory* approach [13-15] was applied to a rather small sample size until saturation of statements was reached. In general, the reported adherence of our population was in line with previously published studies. For instance, the WHO reported that approximately 50% of patients, in general, are adherent [3,4], and a meta-analysis of data on 376,162 patients showed 57% of patients were adherent [17]. This is similar to our results, with 60% reported adherence as shown in Figure 2.

Surprisingly, and in contrast to the study by Manteuffel et al [18], our data indicate an influence of gender. Female participants were more likely to report being adherent than male participants. This influence needs to be considered when adherence solutions are developed.

Figure 2 shows that more academics reported being nonadherent. A reason for that could be higher rates of distrust of academics toward their physician in contrast to nonacademics and maybe a tendency to question the results of the physician [19].

The result in Figure 2 indicates that patients who live by themselves have higher risks of being nonadherent, which is in line with the results of Uchino [20] that social support provides survival advantages to patients with various diseases. Our results confirm the observation of several studies conducted before that reported a drop in adherence over time and especially after the first year of treatment (Figure 2) [21-23].

### Smartphone and Adherence

In accordance with Anderson and Perrin [24] who showed an increase in smartphone use in adults aged 65 years and older from 18% in 2013 to 42% in 2016, our results (Figure 3) show a total smartphone use of 60%. In contrast to female participants, most male participants used a smartphone (90%). This is in line with our other findings that adherence solutions should be gender specific to eliminate the potential problem of a wide demographic spread of the target group (eg, men or women, young or old, and highly or less educated). The results of the study by Anderson and Perrin [24] also confirm our finding displayed in Figure 3 that there was a positive correlation between educational level and smartphone use.

These results and the observation that more academics forgot to take their medicine (Figure 2) strongly suggest the need for creating a smartphone-based adherence solution, especially for academics. It is implied that adherence solutions should always be designed specifically for the customer and individually for the patient. This should not suggest that each smartphone app has to be developed individually for each patient, nor that the software itself should have a unique code. What can be suggested is that each app should be designed to cover the most important adherence factors of different patient groups, that is, the app should include factors such as illness severity and duration, age, gender, and patient’s level of education. In this way, the app can be customized to different patient groups. However, to tailor apps in the future, it does not appear unrealistic to make use of the enormous power of artificial intelligence and machine learning. These technologies continuously analyze the patients and their behaviors while allowing them to improve themselves and adapt according to the gained insights, thereby providing the most individual support.

Furthermore, the cultural background and its impact on technology acceptance should also be taken into account. Several studies have shown the importance of the cultural background for the uptake and use of technology [25-27]. Alagöz et al [28] showed that in contrast to German participants, Polish and Turkish participants significantly increased their acceptance of medical technology as they aged. Alagöz et al [28] hypothesized that this is because of the economic gap and the difference in history between Germany and these countries; this shows that a deeper understanding of the factors underlying technology acceptance, beyond national borders and cultural contexts, is needed. Alsswey et al [29] pointed out that most elderly Arab participants accepted a mobile health apps, which was based and designed on Arab cultural background, and it is important to integrate cultural aspects as well as personal characteristics and experiences into the design process of a mobile health app [29].

If the results shown in Figure 2 are linked to those in Figure 3, it becomes clear that nonadherent patients who live by themselves are also less likely to use a smartphone. Thus, patients who are mostly on their own could be supported with personalized digital adherence solutions, which should include interpersonal relationships. Prochaska and Velicer [30] also highlighted this result using the Transtheoretical Model, developed by Prochaska and DiClemente [31]. They were able to show that individuals must go through six phases of change to change health behavior, and the most promising improvement in computer-based programs is interpersonal contact.

However, looking at the results in Figures 2 and 3, in our cohort, more participants reported being nonadherent after 12 months of NOAC use, but smartphone use among participants was also much higher. This indicates that one could achieve great success with a smartphone-based adherence solution in a long-term medical treatment.

### Adherence Radar

As already shown in several literature reviews, there is a huge bandwidth of new digital adherence solutions in the health care sector with different approaches solving the problem of nonadherence [21,32]. Despite that, none have proven to be successful outside of a clinical study or the pilot phase.

To develop a *new* digital solution targeting that problem, customer feedback seems to be the key. Therefore, we developed a framework to assess the success potential of adherence solutions already in the process of prototyping. Furthermore, the *Adherence Radar* is an analogy and could also provide orientation in a wide range of existing adherence solutions. For this, each main category (as well as related subcategories) should be used to analyze and characterize an already existing or imagined solution. To exemplify, we show how the *Adherence Radar* can be applied to all adherence tools in a holistic manner.

### Adherence Solutions Including Educational Interventions

Educational interventions, as shown by Shah et al [33], can have a great impact on adherence, but as shown in the *Adherence Radar*, several issues need to be considered (category *Diseases and medicine*; Figure 4). Patients stated that they trust their physicians most, and they do not want them to be replaced regarding their disease education by a digital support tool. Furthermore, they stated that they do not necessarily want to know more about their diseases because this would lead to a kind of hypochondria (Main category *Diseases and medicine*; Figure 4). Another point is that education has to be in line with the severity of the underlying illness (Figure 4). We hypothesize that by complying with the *Adherence Radar*, a solution could offer personalized education by the treating physician in the right dose that needs to be defined by the patient.

### Adherence Solutions Using Sensors to Detect Medication Intake

Sensor-based adherence solutions make use of the smartphone to confirm the intake of the medication via visual [34] or ingestible [35] sensors. Such a sensor-based solution could help significantly in severe disease conditions because the patient feels fully cared for. Besides that, these solutions are technically more sophisticated and provide wide flexibility. In the main category *Autonomy* of the *Adherence Radar*, patients stated that they do not want to lose their independence. They worry that the constant digital monitoring promotes the feeling of being sick. An additional aspect that can be addressed using the *Adherence Radar* is one of data privacy and safety. Patients do not want to be monitored in such a close manner and want to act self-determined, points that can be found in the *Adherence Radar* in the main categories *Attitude toward technologies* and *Patient narrative*. The acceptance of a suitable solution is therefore largely dependent on the factors of privacy and the feeling of autonomy.

### Smartphone-Independent Adherence Solutions

The smartphone itself enables some of the adherence solutions to piggyback on a digital device that most of the participants in our study already used. Despite that, other approaches use non–smartphone-based solutions. Examples include robots and other devices as well as a digital pill bottle [36-38]. According to our *Adherence Radar*, bringing in a new device is critical, as the interviewed patients reported that they were already afraid of being dependent on a single digital device. They feared losing their *Autonomy*. It is also possible that the patient feels too much controlled by this additional device, and thus, the self-determination would be lost. In addition, it would touch the *Patient narrative* that increasing costs for digital solutions should be avoided. It needs to be stated that despite all worries, *Technology* itself was widely seen in a positive manner in our study. However, overall, from our point of view, it will be much harder to attain wide acceptance for a non–smartphone-based solution to become routine operation.

### Limitations and Further Research

Our study, to identify factors that make digital solutions successful and generate theses to understand which digital competencies patients aged 65 years and older on anticoagulants should have, to be able to use a digital solution to increase adherence, has a few limitations. The results of the study can only be seen as a snapshot of the topic, which is constantly changing because of the ongoing digitalization. Furthermore, the number of participants seems to be small (N=20), which is because of the chosen approach of the *Grounded Theory*, as the saturation of the statements was reached after 20 interviews. Our study can only be seen as a first step for such a broad and important topic, and further research with a different approach, and therefore a larger number of participants, should be pursued. As further steps in the research, it is necessary to conduct a quantitative confirmation of the factors of the *Adherence Radar* with questionnaires with more than 500 seniors. Additional observational studies are needed to test the use of apps and devices in older people. This could be compared with the group of people aged 55-65 years as the soon-to-be seniors. Thereafter, the vision is to transform the insights of the *Adherence Radar* into a score to compare different adherence solutions clearly.

### Conclusions

The aim of this study was to identify factors that make digital adherence solutions in a real-life scenario successful and how elderly patients can use such a solution. We learned that technology itself is not the problem of a limited uptake and negative view toward digital adherence solutions. As our *Adherence Radar* in Figure 4 demonstrates, the subcategories in the main categories, with the exception of *Technology*, are generally more negatively affected. Here, it becomes clear that digital solutions are partly seen as tools for the younger generation and as gimmickry, but in general, adults aged 65 years and older are open to new technologies as well as digital solutions, and this trend will automatically increase over time, as the aging digital-affine generations will follow. However, it is important that easy access to these new solutions is guaranteed, and these solutions are adapted to the individual patient’s needs. A key element for a successful adherence solution seems to be that it is always designed in a customer-specific manner and uniquely for each patient group. Here, not only gender but also educational background seems to play a role; in addition, the physician-patient relationship is an important factor. The patient must not be made to feel like he/she is losing autonomy and controlled externally, but that he/she is actively and individually supported in his/her medication intake via a digital solution. In our opinion, the smartphone itself seems to be a suitable medium to develop an adequate digital adherence solution for patients because no additional device is needed.

In conclusion, digital adherence solutions can improve the standard of care and help reduce complications of nonadherence. We have shown that there is no universal solution, and tailor-made solutions will be needed.

Our *Adherence Radar* can be a cornerstone in the development of such a solution, for instance, in a design thinking approach, as it helps shed a different light on adherence solutions, in general, and helps people ask the right questions to the right patient.

## Appendix

Multimedia Appendix 1 Consolidated Criteria for Reporting Qualitative Reasearch (COREQ): 32-item checklist.

Multimedia Appendix 2 Interview guideline.

## Abbreviations

COREQ: Consolidated Criteria for Reporting Qualitative Research
NOAC: new oral anticoagulants
WHO: World Health Organization
