# Supplementary material for: Digital Competencies and Attitudes Toward Digital Adherence Solutions Among Elderly Patients Treated With Novel Anticoagulants: Qualitative Study
Source: J Med Internet Res. 2020 Jan 24;22(1):e13077. doi: 10.2196/13077 (PMC7007598; doi:10.2196/13077)
Supplement: Multimedia Appendix 1 [file jmir_v22i1e13077_app1.docx]

# Multimedia Appendix 1.

# Consolidated criteria for reporting qualitative studies (COREQ): 32-item checklist

##

| **No.** | **Item** | **Guide question** | **Response** |
| --- | --- | --- | --- |
| **Domain 1: Research team and reflexivity** | | | |
| Personal characteristics | | | |
| 1. | Interviewer/facilitator | Which author/s conducted the interview or focus group? | Maximilian Herrmann carried out the interviews. |
| 2. | Credentials | What were the researcher’s credentials? *E.g. PhD, MD* | Please see affiliations. |
| 3. | Occupation | What was their occupation at the time of the study? | Please see affiliations. |
| 4. | Gender | Was the researcher male or female? | 1 female, 7 males. |
| 5. | Experience and training | What experience or training did the researcher have? | Most of the researchers have years of experience in clinical and academic research. |
| Relationship with participants | | | |
| 6. | Relationship established | Was a relationship established prior to study commencement? | There was no relationship established to the participants prior the study commencement. |
| 7. | Participant knowledge of the interviewer | What did the participants know about the researcher? *e.g. personal goals, reasons for doing the research* | The participants knew that the interviewer was part of a scientific researcher group at the University of Witten-Herdecke with a common objective of improving the knowledge about how new digital devices can increase adherence in a real-live scenario. |
| 8. | Interviewer characteristics | What characteristics were reported about the interviewer/facilitator? *e.g. Bias, assumptions, reasons and interests in the research topic* | The interviewers provided information in this regard, especially about the interest in knowing the acceptance of digital devices of patients that routinely take a NOAC. |

| **Domain 2: Study design** | | | |
| --- | --- | --- | --- |
| Theoretical framework | | | |
| 9. | Methodological orientation and Theory | What methodological orientation was stated to underpin the study? *e.g. grounded theory, discourse analysis, ethnography, phenomenology, content analysis* | Grounded theory with one data collection episode in form of a semi-structured interview. |
| Participant selection | | | |
| 10. | Sampling | How were participants selected? *e.g. purposive, convenience, consecutive, snowball* | Quota sampling was used for the selection of participants. |
| 11. | Method of approach | How were participants approached? *e.g. face-to-face, telephone, mail, email* | Mail approach. |
| 12. | Sample size | How many participants were in the study? | After 20 participants (5 randomized pilot participants not included), saturation of the statements was reached. 77 participants were identified. |
| 13. | Non-participation | How many people refused to participate or dropped out? Reasons? | 6 participants. The main reason for not participating was that they did not have time or had no interest. |
| Setting | | | |
| 14. | Setting of data collection | Where was the data collected? *e.g. home, clinic, workplace* | At home of the participants. |
| 15. | Presence of non-participants | Was anyone else present besides the participants and researchers? | No, the interviewer and the participant were alone during the interview. |
| 16. | Description of sample | What are the important characteristics of the sample? *e.g. demographic data, date* | Please see Table 1. |
| Data collection | | | |
| 17. | Interview guide | Were questions, prompts, guides provided by the authors? Was it pilot tested? | The authors provided an interview guideline. Interview guideline was previously tested in 5 randomized participants in pilot interviews. |
| 18. | Repeat interviews | Were repeat interviews carried out? If yes, how many? | No, each participant was only interviewed one. |
| 19. | Audio/visual recording | Did the research use audio or visual recording to collect the data? | Each interview was audio recorded with the participant’s permission. |
| 20. | Field notes | Were field notes made during and/or after the interview or focus group? | Yes, field notes were made during the interviews. |
| 21. | Duration | What was the duration of the interviews or focus group? | Average duration of 60-70 minutes. |
| 22. | Data saturation | Was data saturation discussed? | Yes. |
| 23. | Transcripts returned | Were transcripts returned to participants for comment and/or correction? | No. |

| **Domain 3: Analysis and findings** | | | |
| --- | --- | --- | --- |
| Data analysis | | | |
| 24. | Number of data coders | How many data coders coded the data? | Two. |
| 25. | Description of the coding tree | Did authors provide a description of the coding tree? | No. |
| 26. | Derivation of themes | Were themes identified in advance or derived from the data? | Themes were not identified in advance. |
| 27. | Software | What software, if applicable, was used to manage the data? | Audio recorded interviews were coded by using software MAXQDA ® 13. |
| 28. | Participant checking | Did participants provide feedback on the findings? | We will offer it when they were published. |
| Reporting | | | |
| 29. | Quotations presented | Were participant quotations presented to illustrate the themes / findings? Was each quotation identified? *e.g. participant number* | Yes. |
| 30. | Data and findings consistent | Was there consistency between the data presented and the findings? | Yes. |
| 31. | Clarity of major themes | Were major themes clearly presented in the findings? | Yes. |
| 32. | Clarity of minor themes | Is there a description of diverse cases or discussion of minor themes? | Yes. |
