# Supplementary material for: Digital Competencies and Attitudes Toward Digital Adherence Solutions Among Elderly Patients Treated With Novel Anticoagulants: Qualitative Study
Source: J Med Internet Res. 2020 Jan 24;22(1):e13077. doi: 10.2196/13077 (PMC7007598; doi:10.2196/13077)
Supplement: Multimedia Appendix 2 [file jmir_v22i1e13077_app2.docx]

Interview Guideline

The interview guideline is presented to the interviewees in an open manner. Further questions arise from the answers.

The information is subject to § 3 paragraph 6 of the Federal Data Protection Act (BDSG). This means that the results of the survey are anonymized and stored anonymously and therefore no conclusions about your person can be drawn at any time. By agreeing to participate in an interview, you agree that we may collect, store and evaluate your data.

Sociodemographic data:

1. Age and sex?
2. Do you live alone?
3. What do you do occupationally?
4. Indication/illness/duration of treatment/medicine?
5. Do you own and use a smartphone?

Questions about adherence:

1. How do you handle the therapy?
2. How have you been told why you need to take NOAK?
3. Have you previously taken another medicine?
4. What do you know about your disease?
5. How regularly do you take your medicine?
6. Do you have a good feeling about taking your medicine?
7. Do you sometimes forget to take your medicine, and if so, in what situations?
8. How often have you forgotten to take your medicine in the last 4 weeks?
9. How do you get your medicine? Does anything bother you?
10. What would help you to do the therapy better?

Questions about digital solutions:

We will show you 5 short video sequences of possible digital solutions to remind you to not to forget to take your medicine, and ask you to briefly evaluate the different solutions afterwards.

1. What do you think of this solution? What is your impression?
2. What advantages and disadvantages do you see in the solutions?
3. What should be improved so that you would use this solution daily?
4. Do you still have an open thought that you would like to share with me about the solution?

After all video sequences were shown still a final question:

1. Which solution do you like most and why?

Questions about the support by an app:

1. How do you use your smartphone?
2. Do you use email applications or messenger services to communicate?
3. How/where do you shop "electronically" on the Internet?
4. Do you use apps on your smartphone and if so, which ones and why?
5. Tell me about your last use of an app
6. How often do you use apps in your everyday life?
7. Do you use a medical and/or health app?
8. Would you like to have a special medical/health app?
9. How could your motivation to use such an app be increased?

Important: Participation in the study is absolutely voluntary. A refusal to participate or a possible abortion of the interview at any time is without disadvantages for your further treatment. The consent given by you can also be revoked by you subsequently at any time, also without disadvantages for your further treatment. If you revoke your consent, all records made up to that point will be removed from the study and deleted. The retention period of the records and other study documents depends on the retention period of the medical documentation according to the German Civil Code (BGB). In addition, § 630f BGB states that the physician must keep the patient file for a period of ten years after completion of treatment, unless other retention periods apply according to other regulations.
